# Case Study of Endogenous Streptococcal Endophthalmitis in the Critical Care Setting

**DOI:** 10.7759/cureus.16192

**Published:** 2021-07-05

**Authors:** Akshay J Reddy, Nathaniel Tak, James B Martel

**Affiliations:** 1 Opthalmology, California Northstate University College of Medicine, Elk Grove, USA; 2 Ophthalmology, California Northstate University College of Medicine, Elk Grove, USA

**Keywords:** endophthalmitis, antibiotics, cellulitis, sepsis, abscess

## Abstract

We report a rare case of orbital cellulitis and endogenous endophthalmitis, sepsis, meningitis with a brain abscess and a septic knee secondary to Streptococcus pneumonia.

The problems of diagnosis, utility of CT and MRI scanning in the intensive care setting is discussed. The patient was admitted in an obtundated state to the ICU, was noted to have sepsis with blood culture positivity for S pneumoniae. She was noted to have meningitis, a septic knee, a brain abscess and conjunctival injection. CT and MRI scanning did not reveal any ocular or orbital abnormalities. Patient began with a sore throat and knee pain. Despite antibiotic treatment, she became septic with blood culture positivity for S. pneumoniae. She was noted to have knee cellulitis and a brain abscess.

## Introduction

Endogenous endophthalmitis is an inflammatory condition of the intraocular cavities which results from the spread of organisms to the eye from infected sites through the bloodstream. On rare occasions, endophthalmitis may progress to orbital cellulitis with visually devastating outcomes, especially in immunocompromised patients [1]. Endogenous endophthalmitis is a very complex disease and can be hard to detect during the early stage. Despite prompt treatment with appropriate antibiotics, endophthalmitis caused by Streptococcus pneumoniae is associated with a poor visual prognosis [2]. However, it is important that the family physicians and hospitalists be aware of endogenous endophthalmitis because early diagnosis and proper treatment may prevent vision loss in patients.

## Case presentation

A 77- year old East Indian female presented with a one week history of fatigue, high-grade fever and right knee pain. On examination, she was diagnosed with pneumonia and placed on Zithromycin 250mg for five days. Three days later, the patient was rushed to ER and admitted to the hospital for worsening in fever, headaches and confusion. She had a previous history of pneumonia, gastroesophageal reflux disease, hypertension, hyperlipidemia, prolapsed uterus, urethral dilation, vaginal hysterectomy, right total knee replacement and bilateral cataract surgeries. Cerebrospinal fluid was obtained and was noted to have 30-70 white blood cells of which 92% were polymorphonuclear neutrophils (PMNs) with less than 1 glucose and 440 mg/dl of protein, and the gram stain revealed gram-positive cocci. CT scan of the brain was unremarkable. Patient had pneumococcal bacteremia and meningitis and was treated with Levaquin 750mg initial dose, 500mg IV q. 24 hours and Ceftriaxone 2g IV q. 12 hours. 

On the second day of treatment, the patient decreased responsiveness and developed stroke with a systolic blood pressure of 179 mm Hg and a diastolic blood pressure of 69 mm Hg, pulse rate of 85 beats per minute, and core temperature of 99.8 °F. She was transferred to the intensive care unit. On the third day of admission, the patient complained of swelling and redness in the right eye. Ophthalmologic examination showed conjunctival injection, three corneal ulcers and periorbital cellulitis in the right eye. However, the right anterior chamber was well performed and no hypopyon was noted. Her right fundus was not visible ophthalmoscopic ally due to complete opacity. Visual acuity was light perception in the right eye and 25/20 in the left eye. Based upon the clinical examination, endogenous endophthalmitis was diagnosed, although MRI and CT scan of the brain did not indicate any endophthalmitis type of picture. The left eye was normal. 

Intravenous vancomycin treatment was initiated the following day. The consultation was performed because it was believed that the consultation could be used as a means to give the patient broad-spectrum antibiotic coverage in order to treat the patient's pneumococcal bacteremia and meningitis. Additionally, the patient was believed to have had bilateral cataract surgery and their last examination was 3-4 years ago. On day 18, her periorbital edema had nearly resolved. The patient’s condition continued improving with antibiotic drops and ointment. Due to a lack of ophthalmic equipment at the hospital, the patient was brought to the office for a further examination. B-scan ultrasonography of the posterior segment indicated vitreous opacity with no abscess or retinal detachment. Despite intraocular injection and antibiotics drops, the patient had permanent vision loss in the right eye.

## Discussion

Endogenous endophthalmitis secondary to gram positive organisms is relatively uncommon in the East Asian population [3]. It presents diagnostic and therapeutic challenges. The case that we demonstrated was diagnosed by blood cultures and clinical examination. MRI and CT scans in this case do not provide very useful factors but allow us to have a better understanding of the inflammatory process. An early diagnosis and aggressive antibiotic therapy can ameliorate the final course, but the visual outcome still remains poor [4]. However, eye care is often overlooked in the critical care setting or just limited to the ocular surface because treatment is focused on the management of organ failures. In addition to this, it is difficult to detect in the ICU because patients are unable to verbalize symptoms, causing delayed diagnosis and treatment [5].

Therefore, the clinician should be alert to endogenous endophthalmitis whenever a patient with systemic infections complains of ocular symptoms and should have their eyes examined. Endophthalmitis is often a clinical diagnosis and imaging may not always be mandatory, especially for the patient in the intensive care unit [6]. However, they can assist in confirming clinical diagnosis and excluding neoplastic diagnosis. Endogenous endophthalmitis is believed to more commonly affect the right eye due to the direct blood flow from the right carotid artery [7]. Optimal therapy for endogenous endophthalmitis is controversial, especially regarding the role of vitrectomy and intravitreal antibiotics [3]. In this study, it was found that there was a definite improvement in degree of inflammation after intravitreal injection of antibiotics. After careful consideration, the vitrectomy was not performed because there was no benefit for the patient. This was due to the fact that this patient had previously undergone cataract surgery and also due to the fact that the patient was already experiencing a significant decrease in inflammation with the current treatment.

## Conclusions

Given the incidence of exposure to keratopathy causing ocular surface disorder ranging from chemosis and corneal opacification to severe ulceration, it can be difficult for the critical care team to determine when ophthalmological evaluation is required. In the past, it has been a standard to request ophthalmologic consultation when sepsis has been confirmed from blood cultures. However, in the rare circumstance where there is endogenous endophthalmitis, this time may not be optimal. Currently, there is no set standard to request for ophthalmologic consultation, however it can and is generally used as a means to obtain intravitreal antibiotics to treat endogenous endophthalmitis. Prognosis is poor due to the virulence of the infecting organism and its ability to quickly destroy all ocular contents. It affects blood-rich structures such as the retina, choroid, and ciliary body, causing irreversible damage and leading to blindness.
